# Design of Sustainable Aluminium-Based Feedstocks for Composite Extrusion Modelling (CEM)

**DOI:** 10.3390/ma17051093

**Published:** 2024-02-27

**Authors:** José L. Aguilar-García, Eduardo Tabares Lorenzo, Antonia Jimenez-Morales, Elisa M. Ruíz-Navas

**Affiliations:** 1 Powder Technology Group (GTP), Materials Science and Engineering Department, Álvaro Alonso Barba Institute (IAAB), Universidad Carlos III de Madrid, Avda. Universidad 30, 28911 Leganés, Spain; etabares@ing.uc3m.es (E.T.L.); toni@ing.uc3m.es (A.J.-M.); emruiz@ing.uc3m.es (E.M.R.-N.); 2 CIBERINFEC-CIBER de Enfermedades Infecciosas, Instituto de Salud Carlos III, 28029 Madrid, Spain

**Keywords:** aluminium alloy, composite extrusion modelling, powder injection moulding (PIM), sustainable feedstock

## Abstract

Additive manufacturing (AM) has become one of the most promising manufacturing techniques in recent years due to the geometric design freedom that this technology offers. The main objective of this study is to explore Composite Extrusion Modelling (CEM) with aluminium as an alternative processing route for aluminium alloys. This process allows for working with pellets that are deposited directly, layer by layer. The aim of the technique is to obtain aluminium alloy samples for industrial applications with high precision, without defects, and which are processed in an environmentally friendly manner. For this purpose, an initial and preliminary study using powder injection moulding (PIM), necessary for the production of samples, has been carried out. The first challenge was the design of a sustainable aluminium-based feedstock. The powder injection moulding technique was used as a first approach to optimise the properties of the feedstock through a combination of water-soluble polymer, polyethyleneglycol (PEG), and cellulose acetate butyrate (CAB) wich produces low CO_2_ emissions. To do this, a microstructural characterisation was carried out and the critical solid loading and rheological properties of the feedstocks were studied. Furthermore, the debinding conditions and sintering parameters were adjusted in order to obtain samples with the required density for the following processes and with high geometrical accuracy. In the same way, the printing parameters were optimised for proper material deposition.

## 1. Introduction

Aluminium alloys are one of the most important materials in the industry, having multiple applications. Their great relevance is due to various properties, such as excellent strength-to-density ratios and recyclability, thermal and electrical conductivity, and low density (~2.7 g/cm^3^) [1]. The main sectors where aluminium and, most importantly, aluminium alloys are used are the automotive sector, the construction sector, and the aerospace sector. The most common alloys in aeronautical and automotive components are those of the series 2XXX, i.e., Al-Cu-(Mg) 2024, 2224, 2324, and 2524 (damage tolerance), and 7XXX, i.e., Al-Zn-Mg-(Cu) 7075, 7010, 7055, and 7150 (resistance) hardened by ageing [2]. In aeronautics, the 2XXX series aluminium alloys are used with protective coatings on the underwing or in the fuselage due to properties such as slow crack propagation and fatigue strength. In addition, these kinds of alloys usually have poor resistance to electrolytic corrosion due to the presence of precipitates. For this reason, they are coated with alloys with fewer alloying elements that protect them from corrosion, while these aluminium alloys provide resistance [3]. In the automotive industry, parts made from this type of aluminium alloy are usually manufactured by processes such as extrusion. This method is used to manufacture parts with simple profiles. In comparison, the PIM method used in this study as a support tool has some advantages such as the large-scale production of parts with three-dimensional shapes and greater precision. Furthermore, thanks to CEM 3D-printing technology, which is the technique in which the feedstock designed and optimised in this work will be used for the production of 2024 aluminium alloy parts (such as connecting rods or pistons in the automotive industry [4]), it is possible to produce parts with greater freedom in shape and ability to optimise the design. This allows even greater cost savings in producing these parts, in addition to being able to reuse this feedstock. In this context, CEM technology is a cost-effective process without the need for mass production.

There are different processing methods for aluminium alloys depending on the characteristics or properties required for a specific application or component. Most engineering aluminium alloy parts are obtained by conventional manufacturing processes such as casting, forging, and extrusion. While there are many different methods that attempt to produce final aluminium parts with improved properties, they nevertheless present common drawbacks such as limitations in the design of the parts, difficulty in achieving homogeneity, and high energy consumption. Min Wu et al. [5] performed an isothermal uniaxial compression of sintered porous aluminium alloy 2024 at a semi-solid state. The sintered porous materials were prepared from gas-atomised powders by spark plasma sintering (SPS). This study investigated the deformation behaviour of semi-solid powders and simulated the forming process, achieving high levels of relative density in the final parts. In recent studies, Ning Zhao et al. [6] examined the microstructural evolution and mechanical properties of 6082 aluminium alloy parts produced using a novel forging process that integrates forging and solution heat treatment in one operation. Thanks to this advance, these authors were able to obtain aluminium alloys with mechanical properties that are comparable to those produced by conventional processes, with a homogeneous microstructure. Accordingly, this process was adequate for production, application, and industrial promotion thanks to the cost savings entailed. As a novelty, John Victor Christy et al. [7] investigated recycled aluminium alloy composites produced by Stir and Squeeze Casting. The properties, microstructure, and optimisation of the moulded samples were analysed and showed low porosity and high compressive strength for automotive rims.

More recently, although AM of metallic or ceramic materials is currently focused on the prototyping of samples for subsequent production through other techniques, there is a growing number of areas where 3D-printed samples are being used, including energy storage, aeronautics, medical, and environmental applications. Some of these techniques have been used to produce aluminium alloy parts. Processes such as selective laser melting/sintering (SLS/SLM) can produce samples without specific tooling and shorten the design and production cycle, resulting in significant time and cost savings [8]. Agarwala et al. [9] carried out studies combining the liquid-phase sintering/partial melting (LPS) process with SLS, which incorporates the melting of the lower-melting-point material, thus joining the particles of the structural components of the system. Through these techniques, combined with the use of a deoxidiser or atmosphere control, parts with higher density and less geometric distortion (curling or delamination) were produced. Moreover, Das et al. [10] demonstrated the feasibility of manufacturing components for defence applications using the selective laser sintering/hot isostatic pressing (SLS/HIP) technique. The materials processed by those techniques showed good mechanical properties (hardness and tensile strength) equivalent to conventionally processed materials.

However, the processes described above have some drawbacks, such as difficulty in removing the oxide film from the surface of the metal powder, high energy consumption, and elevated cost. Therefore, this study proposes the use of powder injection moulding (PIM), in particular metal injection moulding (MIM), as a first approach to producing samples, in order to obtain final aluminium components with the highest possible densification by Composite Extrusion Modelling (CEM) [11,12]. PIM is a technology that combines polymer injection moulding and metal powders to obtain metal samples with the geometry of the mould used. Through PIM, it is possible to produce highly complex-shaped components with high reproducibility at a low cost, while obtaining good mechanical properties after the sintering step [13]. In this study, the initial characteristics of the powders (particle size, morphology) have been analysed, taking into account previous studies on obtaining bronze 90/10 components by MIM with different powder characteristics in terms of morphology and particle size. Furthermore, thanks to the appropriate selection of the metallic powder content mixed with the binder system, which provides the necessary fluidity for the correct filling of the mould, it is possible to produce metallic samples by injection moulding [14]. Several feedstocks with different optimal solid loadings were produced by combining metal powders and a sustainable binder composed of polyethyleneglycol (PEG) and cellulose acetate butyrate (CAB), taking into account previous studies on the production of zircon feedstocks with this multicomponent sustainable binder system by powder injection moulding [15,16]. The use of alternative sustainable binders, such as the multicomponent system used in this study, is of great importance in reducing the carbon footprint, making it possible to comply with 2030 Agenda Sustainable Development Goal 12 on responsible and environmentally friendly production (ODS 12: Ensure sustainable consumption and production patterns) [17]. The binder components for aluminium feedstocks are usually combinations of PEG, polymethylmethacrylate (PMMA), and high-density polyethylene (HDPE) [18]. Therefore, the use of the PEG and CAB multicomponent binder system for the design and manufacture of an aluminium feedstock, which has already been tested for other materials such as zircon, as mentioned above, is a great challenge. Also, an in-depth study of the complete process (from feedstock optimisation to moulding, debinding, and final sintering stages) was carried out. The rheological properties of the samples were studied, and the different stages of the process were optimised to achieve materials with high densification. To achieve this purpose, one of the most important points is strict control of the thermal debinding to prevent defects from forming in the internal areas of the material. There are studies on the importance of powder and thermal debinding conditions to obtain the highest possible quality in the final part. Dongguo Lin et al. [19] characterised the rheological and thermal debinding properties of BE Ti64 feedstocks. These authors evaluated the critical solid loading, rheological behaviour, and binder decomposition behaviour, concluding that these rheological and thermal debinding properties of feedstock play an important role in the moulding and debinding stage of powder injection moulding (PIM) and can directly determine the quality of the final PIM product.

On the other hand, in the specific case of aluminium alloys of the 2XXX series, such as the 2024 aluminium alloy used in this study, sintering occurs via a transient liquid phase. These liquid phases migrate along the edges of the particles, penetrate the aluminium oxide layer, and diffuse into the powder particles. As a result of the diffusion of the liquid phase and the re-stacking of the aluminium particles, the porosity of the part is reduced. The success of liquid-phase sintering depends on the ability to control the liquid phase [20]. Thanks to the PIM process as a first approach, it was possible to design a sustainable aluminium-based feedstock in a pellet shape and to optimise the parameters required for its use in the CEM additive manufacturing technology. There are previous studies on the use of the PIM process for the manufacture of MAX-phase feedstocks, which were subsequently used in additive manufacturing technology CEM [21].

Within the broad additive manufacturing technologies, one of the most widely used 3D-printing techniques is Fused Filament Fabrication (FFF). In this technology, a filament is extruded through a nozzle while it is being heated to be deposited layer by layer to obtain the desired geometry. As an alternative to filament processes, Composite Extrusion Modelling (CEM) starts from pellets or granulates. Furthermore, CEM offers some advantages compared to FFF, such as the possibility of using a wider binder percentage/quantity range for 3D printing without the need to use a flexible polymeric binder. In addition, the material can be reused if the printing temperatures are selected correctly and the polymer is not degraded during printing. Therefore, by means of CEM, it is possible to print near-net-shape parts using less aggressive conditions, compared to those required in direct powder bed AM technologies, i.e., laser effects on materials, while also allowing a reduction in cost due to savings on material and recyclability [21,22].

## 2. Materials and Methods

### 2.1. Characterisation of Powders and Optimisation of Sintering Cycle

The particle size distribution of the 2024 aluminium alloy pre-alloyed powders (IMR Metal Powder Technologies GmbH, Lind ob Velden, Austria) was determined by laser diffraction using a MasterSizer 2000 (Malvern Instruments, Worcestershire, UK). The powder density was measured with a helium pycnometer (AccuPyc 1330, Micromeritics, Norcross, GA, USA). Additionally, scanning electron microscopy (SEM, TENEO-FEI, Hillsboro, OR, USA) was used to analyse the morphology and microstructure of the cross-section of the powders. To study the thermal behaviour, in order to optimise the sintering behaviour by determining the maximum sintering temperature, the powders were submitted to a thermogravimetric analysis (SETSYS Evolution DTA/DSC, Setaram Instrumentation, Caluire-et-Cuire, France) in a nitrogen atmosphere with a heating rate of 5 °C/min and a final temperature of 800 °C. They were then uniaxially pressed at a low pressure, 340 MPa, in order to reproduce the properties of the brown parts after thermal debinding and before sintering. The green compacts were sintered in a nitrogen atmosphere (1.5 bar) at different temperatures in the interval of 595 to 625 °C for 2 h to design a sintering cycle and to obtain the highest possible densification of the final samples. Scanning electron microscopy and energy-dispersive spectroscopy (EDS) were performed to analyse the microstructure and quality of the sintered samples to determine those similar to the samples obtained after the final sintering stage of the PIM process.

### 2.2. Production of Feedstocks

For the fabrication of feedstocks with different solid loadings from 57 to 70 vol.%, taking into account similar studies on the manufacture of feedstocks of different aluminium alloys [11,18], the Al2024 powders were mixed with a multicomponent binder composed of polyethyleneglycol (PEG), with two different molecular weights of 4000 and 20,000 g/mol, and cellulose acetate butyrate (CAB), with a molecular weight of 30,000 g/mol, in a Haake Polylab QC (Thermofisher, USA). The composition of the pre-alloyed powder is shown in Table 1, which corresponds to the nominal composition of the 2024 alloy, with high surface purity, according to the supplier (IMR Metal Powder Technologies GmbH, Austria), to maintain a uniform interaction with the binder and promote sintering [11,14,23]. The composition of the multicomponent binder selected for the production of feedstocks is detailed in Table 2. In addition, different additives were used as surfactants (stearic acid, SA) and antioxidants (phenothiazine, PTZ). The importance of this binder system lies in the use of PEG, which is water soluble and eliminates the use of organic solvents, which are generally petroleum derivatives and can be toxic, flammable and even carcinogenic, and CAB, which produces low CO_2_ emissions. CAB acts as the backbone in this polymeric binder, and PEG provides fluidity to the mixtures and reduces their viscosity to obtain feedstocks with high solid loadings. Previous studies using zircon feedstocks have shown the good rheological properties of this binder composition for the powder injection moulding process [24]. To study the torque values of different solid loadings in order to propose a critical and optimal solid loading of the feedstock, the same mixer of the mentioned studies was used. For this purpose, the rollers were set to 40 rpm and the torque stabilisation was analysed with a torque rheometer at 180 °C after 0.75 h (45 min) of mixing. In addition, the fluidity of the feedstocks was studied, according to the standard UNE-EN ISO 1133-1:2012 [25]. The test was carried out with a flow meter (CEAST model 6841.000, Pianezza, Italy), in which the different feedstocks were introduced at 180 °C and passed through a hardened steel nozzle with a 2 mm diameter. The weight of the feedstock that went through the nozzle was measured every 0.007 h (25 s). Moreover, the apparent viscosity of the feedstocks was measured in a twin-screw extruder (PolySoft OS-MiniLab 3, Thermofisher, Waltham, MA, USA) using counter-rotating screws varying the speed from 25 to 250 rpm at 180 °C.

### 2.3. Stages of PIM Process

Green samples were obtained by injection of the feedstocks, produced as explained above, in a Bimba Flat 1 injector (AB-400, A.B. Machinery, Peotonoe, IL, USA) at 170 °C with an injection time of 6 s and a pressure of 7 bar. To ensure the structural integrity of the samples, and taking into account previous studies with a similar binder system [26], a two-stage debinding process was performed. The first was a debinding cycle in water at 60 °C with continuous agitation and different immersion times (5 h, 7.5 h, and 10 h) for the removal of PEG from the green parts. Next, the samples were dried in an oven for 1 h at 70 °C. After the binder removal with solvents, an interconnected network of pores was generated. This network facilitates the elimination of CAB during the thermal process preventing defects from forming in the samples, and thus CAB can be removed easily without distorting the geometry of the samples. However, the thermal process must be strictly controlled to prevent defects from forming in the internal zones of the material due to an excessive removal ratio of the backbone.

In this context, the dried samples were then placed in a debinding furnace (GD-DC-50, Goceram, Sweden) at 500 °C for 1 h. Although there are studies on the influence of powder characteristics or the debinding cycle, in this work, a specific study was made on the influence of the atmosphere on the removal of the binder. Accordingly, the thermal debinding was carried out under three different atmospheres, including argon (inert, to prevent the oxidation of the parts during heating as much as possible). Non-oxidising N_2_ and N_2_-H_2_ atmospheres were also used for the thermal debinding stage. However, when using an N_2_-H_2_ atmosphere, hydrides such as AlH_3_ and Al_2_H_6_ are formed. These reactions could cause the partial pressure of H_2_ in the pores to decrease and create a hydrogen gradient between the pores and the external atmosphere. This would slow down the filling of the pores since the internal pressure would be higher, as the solubility of hydrogen is high in liquid aluminium. Refilling the pores with hydrogen does not occur when nitrogen is used since it is not sufficiently soluble in liquid aluminium [27]. The heating and cooling rates were 1 °C/min to ensure proper removal of the backbone polymer (CAB). In order to analyse the percentage of binder removal under the different debinding conditions and to be able to choose the parameters that result in the highest percentage of removal, an analysis was carried out of the dimensional and weight changes after immersion of the parts in water and after the drying cycle. The percentage of PEG and CAB removed as a function of time and atmosphere, respectively, was calculated for each condition, following Equation (1):

(1) $$\%Binder\;removed=\frac{m_{0\;}-m_{s}}{m_{Binder}}\;\cdot 100,$$

where *m*_0_ is the initial mass of the sample; *m_s_* is the mass of the piece after immersion in water, drying, thermal debinding, and sintering; and *m_Binder_* is the total mass of binder in the feedstock according to Equation (2):

(2) $$m_{Binder}=\frac{\%Binder\;\cdot \;m_{0}\;}{100},$$

To verify the complete removal of the binder after the two-stage debinding process following the optimal processing variables, a thermogravimetric analysis was performed on an STA 6000 (STA 6000 PerkinElmer, Waltham, MA, USA) in an argon atmosphere with a heating rate of 5 °C/min and a final temperature of 700 °C.

According to the results obtained for the optimisation of the sintering cycle in low-pressure green samples and those of the thermogravimetric study, the brown samples were sintered in a nitrogen atmosphere at 620 °C for 2 h with heating and cooling rates of 5 °C/min.

The relative densities of the pressed and sintered parts were measured. Moreover, the density of the green samples obtained after injection and after sintering was measured by the Archimedes method to obtain the relative density and the closed porosity, also analysed with ImageJ (Fiji, version 2.35) software.

## 3. Results and Discussion

### 3.1. Characterisation of Powders and Determination of the Sinterability

**Figure 1:**
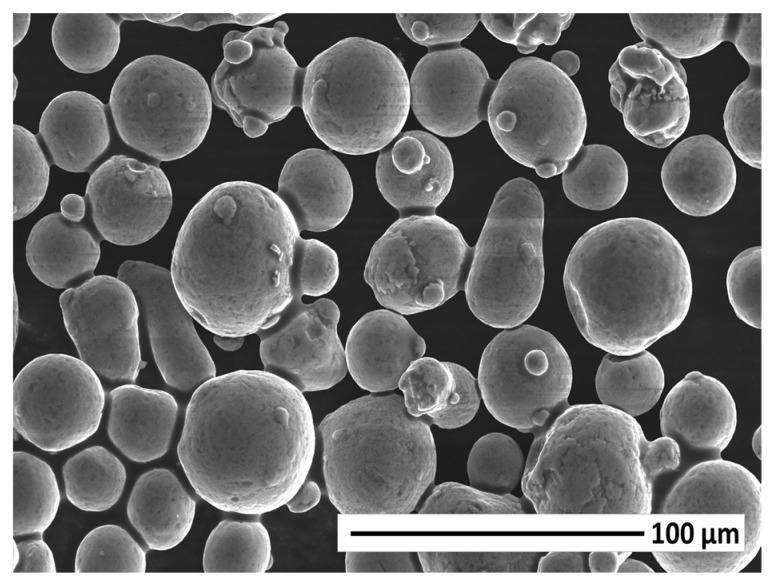
SEM micrograph of the Al2024 alloy powders.

As explained in the previous sections, both the morphological features and the chemical composition of powders must be suitable for the injection process and the subsequent sintering. In this sense, an SEM analysis of the Al2024 aluminium alloy powder particles was carried out. The results can be seen in Figure 1, where the spherical morphology of the particles can be observed. This spherical morphology provides better flowability (the feedstock solid loading can be increased and presents less shrinkage in sintering after injection) [11]. In addition, they are pre-alloyed alloy powders, which favours the homogeneity of the alloy. During the sintering stage, the diffusion process of the alloying elements, the formation of sintering necks, and the liquid phase are more favoured as these elements are more homogeneously distributed within the particles. Therefore, and as will be explained later, this diffusion of alloying elements occurs from the centre of the particles towards the particle boundaries, where the liquid phase is generated.

**Figure 2:**
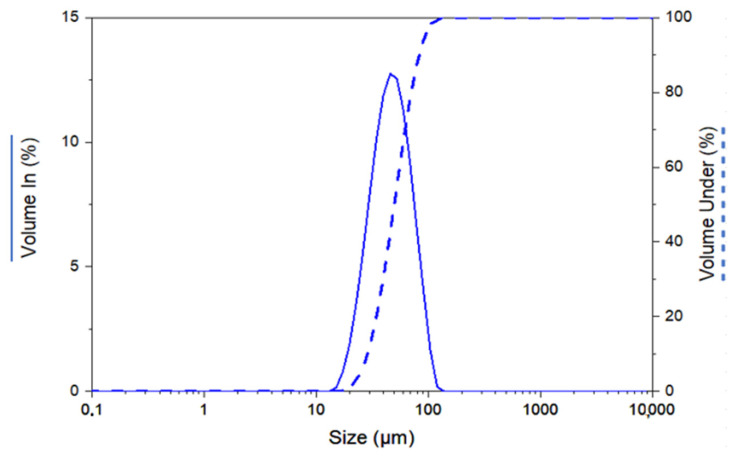
Particle size distribution of the Al2024 powders.

On the other hand, the particle size distribution of the powders was determined with the MasterSizer 2000 analyser. This analysis showed a wide particle size distribution (Figure 2 and Table 3), ranging from 28.89 µm (D_10_) to 82.88 µm (D_90_). A wide particle size distribution of the powders indicates that they are optimal for injection moulding, as they will have a low viscosity and are easy to mould.

**Figure 3:**
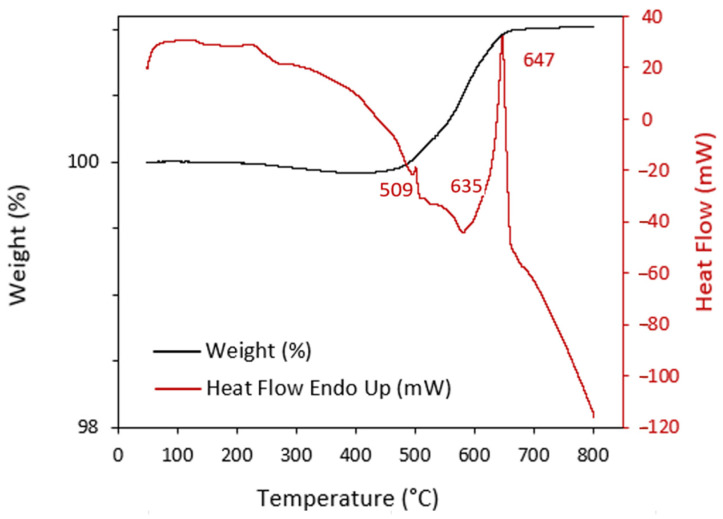
DTA-TG analysis of the Al2024 alloy powders.

In order to perform the final sintering of the parts after thermal debinding, it is important to analyse the thermal behaviour, with the melting temperature of the feedstock metal-based material, among others. A DTA-TG analysis was carried out with the 2024 aluminium alloy powders to obtain the heat flow curve of this material (Figure 3) and thus be able to establish an optimal sintering temperature. In this test, it is evident that there are two endothermic peaks, a small one at 509 °C and a big one at 647 °C, corresponding to the melting point of this pre-alloyed powder with the onset temperature at 635 °C. The occurrence of a phase transition during heating was suggested by the endothermic peak at 509 °C, which corresponded to the production of a liquid phase in aluminium by a eutectic reaction. Therefore, the solidus temperature of the powders of this 2024 aluminium alloy was 509 °C, consistent with analyses from previous studies of this alloy [28]. In addition, a mass gain can be seen at 500 °C due to oxidation of the aluminium powders.

**Figure 4:**
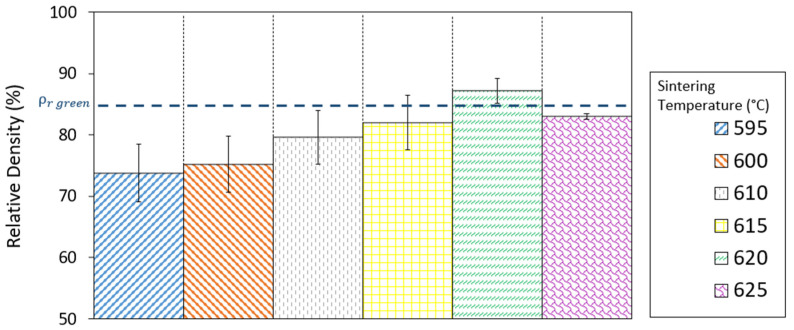
Relative density of Al2024 parts sintered at different temperatures from 595 to 625 °C.

Considering the results shown above regarding the thermal behaviour of the powders, and to further examine the sinterability of samples after debinding (brown samples), the powders were uniaxially pressed at low pressure (340 MPa) and sintered in a nitrogen atmosphere (1.5 bar) at a temperature range of 595–625 °C for 2 h. The theoretical density of the 2024 aluminium alloy, with a value of 2.8 g/cm^3^, was obtained by the measurement of the powders’ densities in a helium pycnometer. Green and sintered densities were measured by the Archimedes method. The relative green density of these low-pressed powders was 84.2% (Figure 4, blue dotted line) and that of sintered samples at different temperatures are shown in Figure 4.

**Figure 5:**
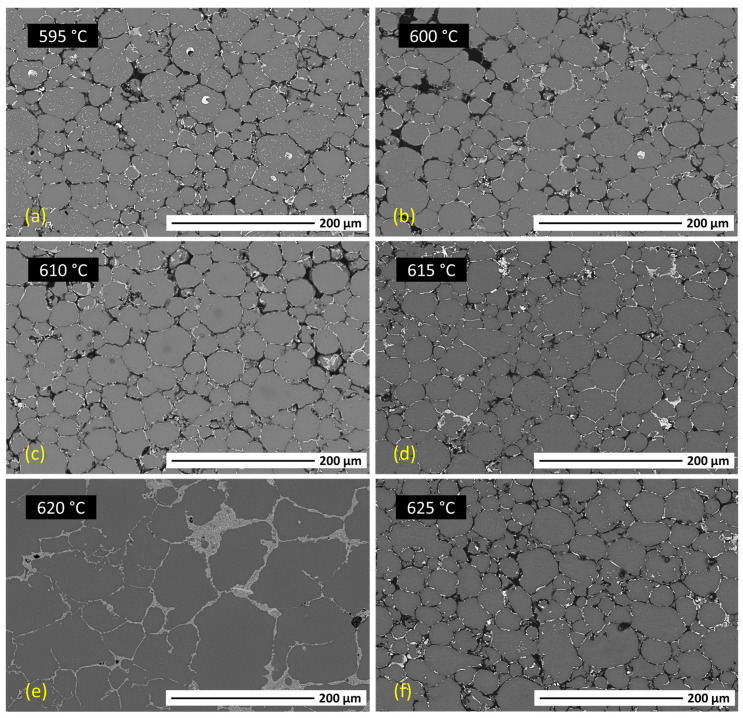
SEM micrographs of the cross-section of Al2024 parts sintered at different temperatures of (**a**) 595 °C, (**b**) 600 °C, (**c**) 610 °C, (**d**) 615 °C, (**e**) 620 °C, and (**f**) 625 °C.

Density values obtained for the pressed and sintered parts were markedly low compared to the theoretical density of the 2024 aluminium alloy, due to the presence of a high level of porosity (see Figure 5) and swelling of the parts. However, it can be observed that the only parts that densified by obtaining a relative density higher than the green density were those sintered at 620 °C, with a relative density value of 87.2%. These parts suffered the least distortion and shrank (2.39 ± 0.19% on the X axis and 1.31 ± 0.31% on the Y axis). Because of the need to improve this, some studies have focused on the benefit derived from the addition of small amounts of tin to the composition [29,30]. As can be seen, the highest relative density after sintering was obtained with the sintering temperature of 620 °C. Density decreased again in samples when the sintering temperature was increased to 625 °C, showing higher porosity. This indicates that the optimal sintering temperature was overcome.

SEM analysis of the cross-sections of the Al2024 samples after sintering at 595, 600, 610, 615, 620, and 625 °C was carried out to analyse the sintering process and the porosity of the samples (Figure 5).

As a pre-alloyed aluminium alloy powder, a supersolidus sintering process was produced. The relative density increased with increasing sintering temperature, as can be seen in the micrographs obtained, which show a decrease in porosity with increasing temperature. It should be noted that this pre-alloyed aluminium powder is always covered by a thin oxide layer (Al_2_O_3_). This implies that the liquid phase generated during sintering is prevented from penetrating through this oxide layer. Therefore, in samples sintered at low temperatures, the formation of the liquid phase was not sufficient to achieve high densification, and porosity was generated. However, at the sintering temperature of 620 °C, a network of the former liquid phase was observed. This liquid phase was generated from the Cu- and Mg-rich precipitates in the particle boundaries, as verified by subsequent EDS analyses. These eutectic phases promoted the densification and re-stacking of the particles and a remarkable decrease in porosity, as in other studies on the sintering of aluminium powders [28,31,32].

In contrast, increasing the sintering temperature up to 625 °C again resulted in an increase in porosity. During supersolidus liquid-phase sintering, it is important to obtain an optimal volume of the liquid phase, which is conditioned by the sintering temperature and the characteristics of the powder. An excess of liquid phase results in the loss of part shape and can even lead to pore growth [31,33,34]. Therefore, compared with the sample sintered at 620 °C, the sample sintered at 625 °C suffered shape distortion and pore growth. Thus, the relative density of the sintered part at the highest temperature was reduced, which corroborates the results obtained in Figure 4.

**Figure 6:**
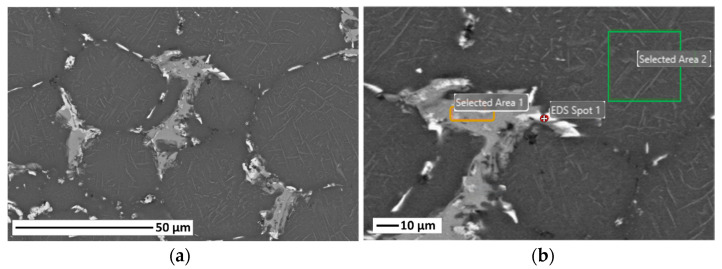
(**a**) Micrograph of AA2024 sintered at 620 °C; (**b**) detailed micrograph of the grain boundaries.

In addition, EDS analysis of the samples sintered at different temperatures was also carried out. This semi-quantitative analysis showed that the distribution of the alloying elements (Al-94.4 wt.%, Mg-1.8 wt.%, Cu-3.8 wt.%) was homogeneous for all of them in accordance with the theoretical composition of Al2024 and the pre-alloyed powders. In particular, several analyses were performed in samples sintered at 620 °C, as seen in Figure 6. Figure 6b shows an example of one of the analyses in a selected area in the grain boundaries, which could correspond to the former liquid phase rich in copper and other alloying elements such as Mg, Mn, or Fe. Table 4 shows the results obtained in the EDS analysis seen in Figure 6b.

### 3.2. Solid Loading Determination and Rheological Properties of the Feedstocks

**Figure 7:**
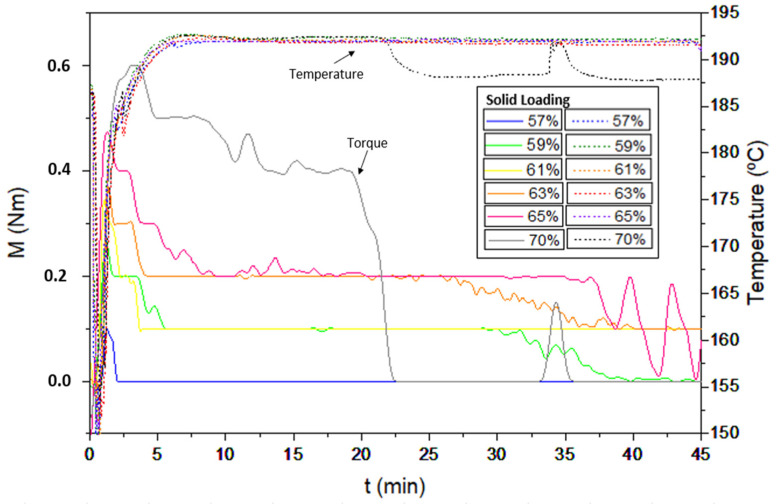
Variation in torque and temperature with time for PEG/CAB feedstocks with different solid loadings of Al2024 from 57 to 70 vol.%.

There are numerous studies on the employment of different percentages of solid loading according to the powder properties as well as different binder systems for the manufacture of feedstocks and parts by powder injection moulding [11,18,35]. In these studies, the solid loading varies from 50 vol.% to 65 vol.%. It is important to establish the critical solid loading because during the mixing step, an excess of binder can lead to defects such as burrs or crumbling during the debinding process. On the other hand, an excess of powders could lead to defects such as porosity or high viscosity [36]. In this study, for the manufacture of the feedstocks and to be able to establish the value of the critical solid load, different solid loads from 57 to 70 vol.% were selected to study their rheological behaviour by means of the torque rheology of the feedstocks. Initially, the binder system was poured into the mixing chamber to melt for 0.166 h (10 min). After that, the amount of powder, depending on the solid content of each feedstock, was inserted into the chamber. As can be seen in Figure 7, during the mixing of all the feedstocks, there was an increase in torque when closing the mixing chamber, which progressively decreased and stabilised over time at a relatively low torque value while the mixture was homogenising. In the case of the feedstock with 65 vol.% of solid loading, the torque value was not constant after the mixing time due to the excessive amount of solid loading in the mixture. This feedstock was established as the critical solid loading. When the torque is unstable, it means that the powder loading has surpassed the critical value. For mixtures above this volume of solid loading, an inhomogeneous feedstock was created in which the binder could not accommodate more powder and did not mix well with it. It is possible to observe a final decrease in the torque value at the end of the mixing time as in the case of the feedstock with 70 vol.% of solid loading.

According to these results, feedstocks with 61% and 63 vol.% of metallic powders were established as the optimal solid loadings of those studied. These feedstocks had better rheological behaviour as they presented an optimal mixture and a better homogeneity between powder and binder for the injection process. However, the processability of the feedstocks obtained and the optimal solid loading, which is 2–5% less than the critical loading, must be taken into account [24,37,38]. Although the temperature was set at 180 °C, the average temperature reached values of around 192 °C for the production of all the feedstocks, due to the internal friction between metallic powder particles and binder during the mixing process.

**Figure 8:**
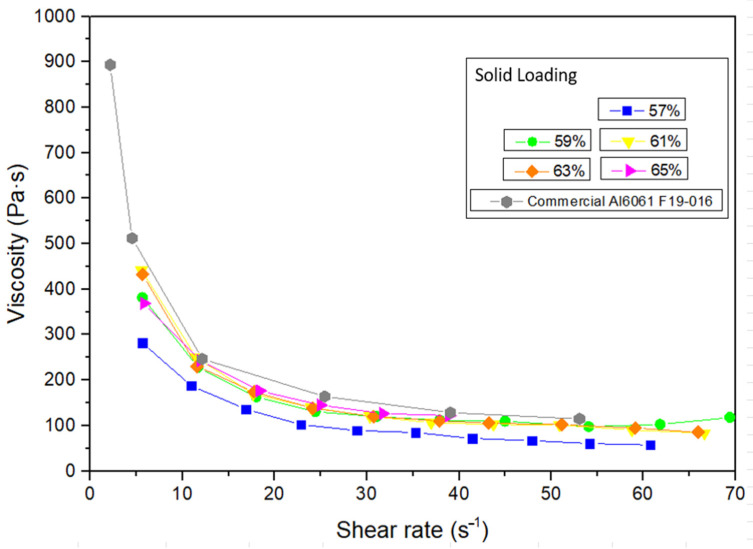
Apparent viscosity at different shear rates of the PEG/CAB feedstocks with a solid loading of Al2024 from 57 to 65 vol.% and commercial Al6061 F19-016.

**Figure 9:**
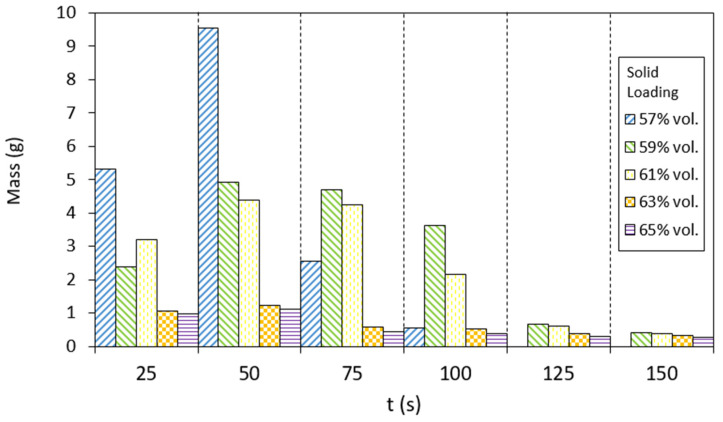
Variation in the fluidity of the PEG/CAB feedstocks with a solid loading of Al2024 from 57 to 65 vol.%.

In addition, the apparent viscosity of the feedstocks produced was analysed. As seen in Figure 8, all mixtures showed pseudo-plastic behaviour, whereby with increasing shear rates, the viscosity decreases. It can also be observed that by increasing the solid loading of the feedstocks, the viscosity increases slightly. PEG/CAB feedstocks were compared with a commercial feedstock of Al6061 F 19-016 (70 vol.%), showing slightly lower viscosity values due to their lower solid loadings. Although viscosities increase slightly with higher solid loadings, they always remain below the recommended viscosities for powder injection moulding (1000 Pa·s) [39]. The fluidity of the feedstocks was also studied, as can be seen in Figure 9. With this test, the pseudo-plastic behaviour of the feedstocks was again observed, where the flow capacity of the feedstocks decreased as the solid load in the mixture increased. It was observed that the feedstock with 57 vol.% of solid loading flowed too fast in the first seconds of the test, confirming that the mixture still allowed more solid load admixed, while the feedstock with 65 vol.% of solid loading scarcely presented any variation in fluidity throughout the test. It is important to obtain a balanced mixture between binder and powder, in which more homogeneous regions are created and the viscosity is lowered, providing the material with the necessary fluidity for the injection step for the PIM process [40]. Therefore, considering the results obtained for the rheological properties studied with the different solid loadings, the feedstocks with 61 vol.% and 63 vol.% of solid loading were selected as the optimal solid loading.

### 3.3. Processing of the Injected Samples: Debinding and Sintering

As explained in the previous section, the optimal solid loading is 2–5% less than the critical loading, and the processability of the feedstocks obtained must be taken into account. Since it was very difficult to obtain injected parts using the feedstock with 63 vol.% of solid loading, as it had a very high solid loading, the 61 vol.% feedstock was selected as the optimal solid loading for the production of green parts and their subsequent debinding and sintering. The aluminium alloy feedstocks manufactured in the above-mentioned studies usually have a much smaller particle size (in this work, the D_50_ of our aluminium alloy powders was 45 µm) for the production of parts by the PIM process as this greatly facilitates the process. However, our work used a commercial 2024 aluminium alloy powder that was much coarser, which provided optimal solid loadings similar to those obtained in other studies with non-commercial powders designed to be finer.

Furthermore, compared to previous studies using this combination of PEG and CAB in a multicomponent binder system for obtaining feedstocks as MAX phases [38], the optimal solid loading was around 52 vol.%, whereas, in our study, the optimal solid loading was 61 vol.%. In order to know the amount of binder removed during the different debinding processes, the mass of nine samples was measured for each condition before and after debinding in water and also before and after thermal debinding. By comparing the results obtained from the masses of the samples before and after the different hours of water immersion and before and after thermal debinding in the different atmospheres, the optimum conditions of water debinding and subsequent thermal debinding were selected. Thus, taking into account previous studies [38], the two steps with the most favourable debinding conditions for this binder removal were a solvent debinding cycle in water at 60 °C with continuous agitation and an immersion time of 5 h for the removal of PEG from the green parts, followed by a thermal debinding for 1 h at 500 °C to eliminate the rest of the binder components in an argon atmosphere. An analysis of the mass variation was performed throughout the debinding cycle and, thanks to Equations (1) and (2), the amount of polymer removed was calculated. A 90 wt.% polymer removal was achieved after this two-stage debinding. For any other condition, the achieved binder removal was less than 90 wt.%. It is important to attain complete binder removal or as much as possible in the debinding stage in order to achieve high densification of the final parts in the sintering step [41]. Furthermore, an inert argon atmosphere, which reduces or prevents the formation of an Al_2_O_3_ layer, has been found to be more effective in eliminating this kind of polymer than any other atmosphere [35]. Nevertheless, previous studies, like those of Z. Y. Liu et al., have shown that for the sintering of aluminium alloy 6061 obtained by injection moulding, a nitrogen atmosphere is more effective [30]. That is why an argon atmosphere was not used for the sintering stage.

**Figure 10:**
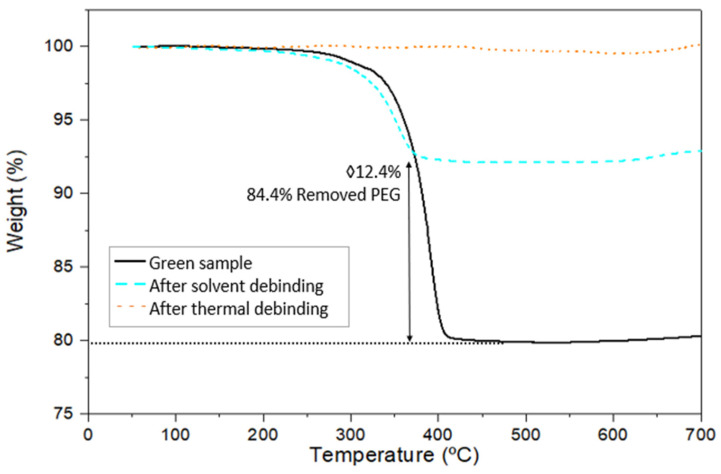
TGA analysis of PEG/CAB feedstock with a solid loading of 61 vol.% of Al2024 for the green simple (black line), after solvent debinding (blue dashed line), and after thermal debinding (brown dashed line).

To verify these results from mass losses of the feedstock and confirm the removal of the polymeric binder, a thermogravimetric study of the green parts after debinding in water and after thermal debinding was carried out. Figure 10 shows the different thermogravimetric analyses for the green and brown parts for feedstock with 61 vol.% Al2024. The first analysis (black line), corresponding to the green part, shows a mass loss starting at 260 °C and ending at 405 °C; this mass loss reaches 79.9 wt.% related to the degradation of 39 vol.% of the binder in the feedstock. The second analysis (dashed blue line) belongs to the sample after PEG removal through solvent debinding. It can be seen that the part loses 12.4% less mass compared to the green sample, which corresponds to the 84.4 vol.% of the PEG removed. Finally, the third analysis (dotted brown line) shows that after the thermal cycle, all the polymeric binder has been removed from the brown parts, including the residual PEG and all the CAB from the brown part. Therefore, in accordance with the results obtained, no mass loss is expected to occur during the third analysis.

As previously seen by the study of the mass loss during the removal of the binder with Equations (1) and (2), a 90 wt.% polymer removal was obtained. However, the oxidation of the samples and the consequent weight gain during debinding may justify that a complete removal of the binder was not obtained by these equations. Thus, the thermogravimetric study of the samples was carried out in the STA. As can be seen in Figure 10, the STA test shows a complete removal of the binder, giving a more precise value of the polymer removed. These results justify the selected thermal debinding cycle of 1 h at 500 °C for the removal of CAB and the rest of the polymers from the binder, PTZ and AE, considering that the PEG polymer was previously removed by dissolution in water. In addition, after the debinding process, all the samples maintained their structural integrity and no significant defects were observed in the brown parts.

As described above, thanks to the pressing and sintering of Al2024 powder samples and their subsequent analysis, and taking into account previous studies on the sintering of different aluminium alloys [30,32], a temperature of 620 °C was selected as the optimal sintering temperature. Therefore, the same sintering cycle was employed for the parts produced from 61 vol.% Al2024 feedstock obtained by PIM.

**Figure 11:**
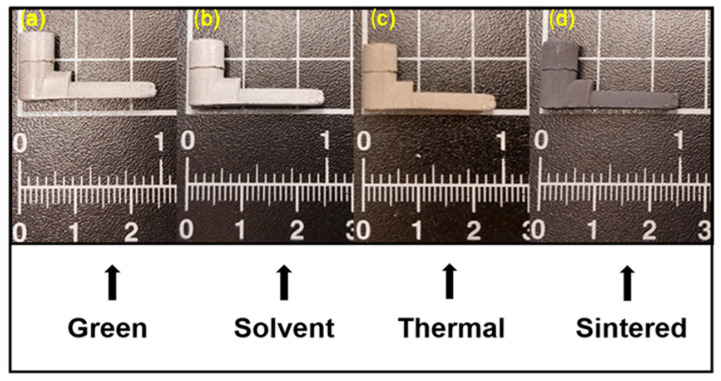
Injected samples of feedstock with a solid loading of 61 vol.% of Al2024 obtained at different stages: (**a**) green part, (**b**) part after solvent debinding, (**c**) part after thermal debinding, and (**d**) sintered part.

Figure 11 shows an example of parts after different stages of the PIM process, i.e., after injection (Green), debinding (Solvent and Thermal) and sintering (Sintered). These images show that all parts maintained their structural integrity even after the debinding and sintering steps. This indicates that both processes were carried out under optimal conditions to obtain good-quality parts without crumbling.

Table 5 shows the results of relative density and closed porosity obtained in green parts after the injection process, calculated with the Archimedes method. To obtain the relative density of green parts, the theoretical green density of the feedstock was calculated using the rule of mixtures. Table 5 also shows the relative density of parts after sintering. This value was obtained using the density of the Al2024 powders, measured with the helium pycnometer, as theoretical density. As can be seen, these relative density values are similar to those achieved in the pressed and sintered parts (see Figure 4), validating the PIM process for the optimisation of the debinding and sintering stages in order to obtain densified samples. The parts obtained by PIM, as well as the pressed and sintered parts, underwent shrinkage (3.11 ± 0.21% on the X axis and 1.71 ± 0.24% on the Y axis).

This study of the optimisation of the debinding and sintering parameters of Al2024 aluminium alloy feedstocks confirms their viability for production by the PIM process. Parts of this alloy by means of 3D CEM printing technology are in production, taking into account previous studies with similar binder systems for the extrusion-based additive manufacturing of Ti_3_SiC_2_ and Cr_2_AlC MAX phases [21].

## 4. Conclusions

2024 aluminium alloy parts were produced by powder injection moulding. In particular, the parameters for the production and processing of sustainable feedstocks of an Al2024 alloy with a multicomponent binder system of PEG and CAB (in a ratio of 70–30 vol.%) were determined using the PIM process. This makes it possible to comply with 2030 Agenda Sustainable Development Goal 12 on responsible and environmentally friendly production (ODS 12: Ensure sustainable consumption and production patterns). For that purpose, the optimal solid loading of the feedstocks was determined. Moreover, feedstocks produced with optimal processing conditions showed good rheological behaviour, with low torque values, low viscosity, homogeneous mixing, and pseudoplastic behaviour. The final optimal solid loading selected contained 61 vol.% of Al2024 powders. The two-step debinding process was optimised and controlled with thermogravimetric analysis for total binder removal and consisted firstly of a solvent debinding at 60 °C for 5 h and a subsequent thermal debinding at 500 °C for 1 h in an argon atmosphere. The sintering step was also optimised and determined at 620 °C for 2 h in a nitrogen atmosphere. For this purpose, SEM analysis of the cross-sections of Al2024 samples pressed and sintered at different temperatures was carried out. The sintering conditions were employed for injected samples, achieving a relative density of 87.2% with a shrinkage of 2.39 ± 0.19% on the X axis and 1.31 ± 0.31% on the Y axis. Similarly, the 61 vol.% Al2024 feedstock parts reached a relative density of 83.7% with a shrinkage of 3.11 ± 0.21% on the X axis and 1.71 ± 0.24% on the Y axis. This relative density value obtained in sintered parts can be improved by taking into account previous studies focusing on the benefit derived from the addition of small amounts of tin to the composition. Thanks to the optimisation of the feedstock carried out in this study, current work is now being developed using CEM for the production of parts of Al2024 alloy. This technology will enable cost savings compared to other laser additive manufacturing technologies or technologies using filaments.

## Figures and Tables

**Table 1:** Composition of the aluminium alloy.

Element	Si	Fe	Cu	Mn	Mg	Cr	Zn	Ti	Al
wt.%	0.10	0.13	4.67	0.55	1.73	<0.01	0.11	<0.01	balance

**Table 2:** Composition of the multicomponent binder system used.

Binder	PEG	CAB
Vol.%	70	30
Molecular weight (g/mol)	4000	30,000
	20,000	
Supplier	Sigma-Aldrich	Eastman

**Table 3:** Mean particle sizes of Al2024 alloy powders, obtained from particle size distribution analysis by laser.

D_10_ (µm)	D_50_ (µm)	D_90_ (µm)
28.89 ± 0.01	49.85 ± 0.02	82.88 ± 0.03

**Table 4:** EDS analysis in different zones of the detailed micrograph for the Al2024 parts sintered at 620 °C.

Element	Weight %	Error %
EDS Spot 1		
Mg	1.6	11.4
Al	60.3	7.2
Mn	1.2	15.5
Fe	1.2	16.5
Cu	35.6	3.0
Selected Area 1		
Al	71.8	6.0
Mn	7.5	4.3
Fe	9.4	4.1
Cu	11.3	5.0
Selected Area 2		
Mg	2.0	6.8
Al	93.9	4.0
Mn	0.6	25.6
Cu	3.5	9.0

**Table 5:** Relative density and porosity of green parts and sintered parts from feedstock with a solid loading of 61 vol.% of Al2024.

Feedstock	Green Parts		After Sintering	
Al2024 61 vol.%	**ρrel (%)**	**Closed Porosity (%)**	**ρrel (%)**	**Closed Porosity (%)**
	79.1 ± 1.5	20.9 ± 1.5	83.7 ± 1.7	16.3 ± 1.7

## Data Availability

Data are contained within the article.
